# Patients’ Needs for Care in Public Mental Health: Unity and Diversity of Self-Assessed Needs for Care

**DOI:** 10.3389/fpubh.2016.00022

**Published:** 2016-02-17

**Authors:** Tanja Bellier-Teichmann, Philippe Golay, Charles Bonsack, Valentino Pomini

**Affiliations:** ^1^Institute of Psychology, University of Lausanne, Lausanne, Switzerland; ^2^Community Psychiatry, Department of Psychiatry, Lausanne University Hospital (CHUV), Lausanne, Switzerland

**Keywords:** ELADEB, needs assessment, severe mental illness, public mental healthcare, patient-centered practice, care community

## Abstract

**Purpose:**

Needs assessment is recognized to be a key element of mental health care. Patients tend to present heterogeneous profiles of needs. However, there is no consensus in previous research about how patients’ needs are organized. This study investigates both general and specific dimensions of patients’ needs for care.

**Methods:**

Patients’ needs were assessed with ELADEB, an 18-domain self-report scale. The use of a self-assessment scale represents a unique way of obtaining patients’ perceptions. A patient-centered psychiatric practice facilitates empowerment as it is based on the patients’ personal motivations, needs, and wants. Four seventy-one patients’ profiles were analyzed through exploratory factor analysis.

**Results:**

A four-factor bifactor model, including one general factor and three specific factors of needs, was most adequate. Specific factors were (a) “finances” and “administrative tasks”; (b) “transports,” “public places,” “self-care,” “housework,” and “food”; and (c) “family,” “children,” “intimate relationships,” and “friendship.”

**Conclusion:**

As revealed by the general factor, patients expressing urgent needs in some domains are also more susceptible to report urgent needs in several other domains. This general factor relates to high versus low utilizers of public mental healthcare. Patients also present specific needs in life domains, which are organized in three dimensions: management, functional disabilities, and familial and interpersonal relationships. These dimensions relate to the different types of existing social support described in the literature.

## Introduction

Needs assessment is recognized to be a key element of mental health care. People with severe mental illness often have a multifaceted combination of clinical and social needs. Scientific literature has not only shown that staff assessment and self-assessment of patients’ needs diverge (1–7) but has also revealed that authors do not agree on a unanimous structure of needs (8–11). These results reveal the difficulty in identifying a stable structure of patients’ needs that would synthesize the way patients’ psychosocial needs are organized.

Indeed, patients’ needs profiles are individual and context-dependent. Therefore, having only one structure covering the whole variety of profiles seems hard to achieve. Another explanation for these disparities in the dimensions of needs found in the literature may rely on the statistics used in the studies, and more precisely on the limitations of the statistics implemented in the factor analyses (12–14). When traditional methods for factor rotation fail to reveal clear and interpretable solutions, the exploration of more complex structures presenting both general and specific factors needs to be considered (15, 16). For instance, recent rotation methods, such as bi-geomin, also referred as bifactor exploratory factor analysis (EFA) allows the simultaneous extraction of a general factor and specific factors that account for the unique influence of specific domains over the general factor (14).

Finally, the type of needs assessment instruments used could also lead to various results. Most of the studies measuring the structure of patients’ needs used the Camberwell Assessment of Need (CAN) (17). This tool assesses the needs of people with severe and enduring mental illness. It covers a wide range of health and social needs and includes staff and user assessment. Various versions adapted to specific types of populations have been developed including a simplified patient self-assessment version [CAN Short Appraisal Schedule – Patient version (CANSAS-P)] (18). The CAN is generally considered useful and feasible for the standardized assessment of patients in routine mental health care. Despite its good psychometric qualities, its wide diffusion in the mental health services and the numerous citations about it in the scientific literature, this instrument is not exempt from criticism. This tool allows a simultaneous self and staff evaluation that facilitates the clinical comparison of results. Nevertheless, there are two shortcomings with this method: it involves plenty of staff time in eliciting the patients’ ratings and, more importantly, it potentially implies staff filtering of the patients’ perspective (19). Self-assessments of needs may therefore be influenced by the staff’s perception of patients’ actual needs. Moreover, Trauer et al. (18) showed that chronic patients with psychiatric disorders experience difficulties even when completing simple questionnaires, such as the CANSAS-P. Almost a third of the patients reported difficulties in understanding how to fill out this questionnaire. Even if it appears simple to use, the CANSAS-P seems therefore unsuitable for assessing chronic patients who present cognitive, verbal, or language deficits. These limitations may thus be a possible explanation for the divergent needs structures obtained in the literature, especially when chronic patients are involved.

In response to these methodological and statistical limitations, we conducted a study with a more recent self-assessment scale and using a recently published statistical approach. In order to bypass the aforementioned limitations of the CANSAS-P with cognitively impaired patients, we used a different kind of self-reported tool: the difficulties and needs self-assessment tool (ELADEB). The assessment is achieved through a Q-Sort method facilitating a systematic quantitative evaluation of the patients’ difficulties and needs by sorting cards. For a detailed discussion on the benefits of the Q-Sort approach, refer to Bellier-Teichmann and Pomini (20). This instrument aims at (1) avoiding clinicians’ interferences regarding patients’ perceptions and (2) clarifying the concept of need by defining and measuring the urgency of needs and therefore a timeline for desired interventions. We hypothesized that the bifactor EFA could give a better factorial solution of patients’ needs. In the study reported here, we analyzed data from 471 profiles of patients coming from three mental health centers. Our purpose was to observe whether the structure of needs, using this different self-assessment scale as well as recent statistical method, was similar to the previous results obtained in the literature or whether it leads to a new model.

## Materials and Methods

### Participants

This multicentric study was approved by the Swiss National Health Service Research Ethics Committee. Patients were informed about the confidentiality of data and their right to withdraw from participation at any time. Written, informed consent was obtained from all patients. The difficulties and needs self-assessment tool (ELADEB) was administered to three groups of patients as a part of routine clinical assessments of three different mental health centers in the Department of Psychiatry at the University Hospital in Lausanne, Switzerland: (1) hospitalized patients participating in a case management program preparing their discharge from the hospital (*n* = 104); (2) patients taking part in an evaluative and treatment rehabilitative program for outpatient care (*n* = 215); and (3) patients in sheltered workshops and supervised housing (*n* = 152). The overall sample includes 471 patients (see Table 1). Participants were recruited between June 2008 and January 2013 through referrals from health professionals at each location. Assessments were conducted by one mental health professional (psychologist, nurse, or occupational therapist) who had received standardized training prior to the study. The interviews lasted about an hour. For standardization purpose and in order to avoid investigators’ interferences regarding patients’ perceptions, the investigator and the patient were asked to remain silent during the card-sorting procedure: patients were informed that a structured discussion would be conducted right after the ratings were recorded. This discussion allowed the patient and the investigator to specify and freely discuss each need without influencing ratings. Inclusion criteria were (a) being between 18 and 70 years of age, (b) meeting ICD-10 criteria for a psychiatric diagnosis, and (c) having sufficient French language skills to understand the items. Exclusion criteria were (a) incapacity of discernment, (b) diagnosis of organic mental disorders, and (c) presence of acute symptoms impeding assessment with the ELADEB. ICD-10 diagnostics were provided by the treating psychiatrists who were not present during the assessment.

**Table 1:** **Participants’ sociodemographic characteristics**.

Characteristic	
Mean age (years)	40 (SD = 5.6)
Gender [*n* (%)]	
Female	228 (48.4%)
Male	243 (51.6%)
Marital status [*n* (%)]	
Single	315 (66.9%)
Married	80 (17%)
Other	76 (16.1%)
Living situation	
Independent apartment	125 (26.5%)
With partner or family	165 (35%)
Supervised housing	173 (36.7%)
Other	8 (1.8%)
Schooling	
No schooling	57 (12.1%)
Compulsory school	147 (31.2%)
Apprenticeship	111 (23.6%)
High school, diploma, and secondary school	47 (10%)
Other	109 (23.1%)
Occupation	
No professional activity	172 (36.5%)
Supervised workshops	150 (31.8%)
Training (apprenticeship, training college, and secondary schools)	18 (3.8%)
Other	131 (27.9%)
Diagnosis categories (ICD-10)	
Mental disorders due to psychoactive substance use (F1)	30 (6.4%)
Schizophrenia (F2)	159 (33.8%)
Mood disorder (F3)	110 (23.4%)
Neurotic and anxiety disorder (F4)	42 (8.9%)
Behavioral syndromes (F5)	24 (5.1%)
Personality disorder (F6)	40 (8.5%)
Mental retardation (F7)	40 (8.5%)
Disorders of psychological development (F8)	21 (4.5%)
Unspecified mental disorder (F99)	5 (1.1%)

### Measures

**Figure 1:**
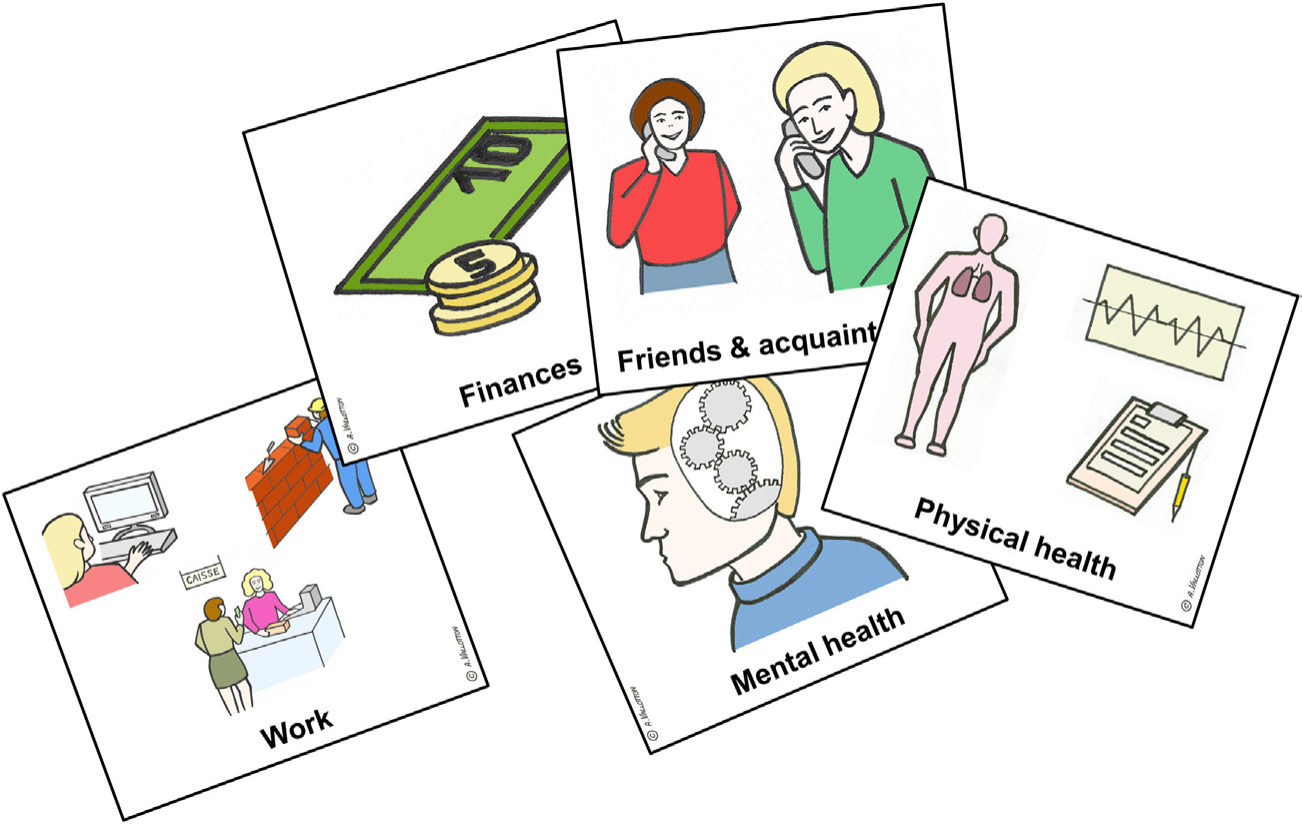
**Example of the Q-sort cards used in the ELADEB**.

The ELADEB has been used to measure patients’ needs. In order to bypass the limitations of traditional questionnaires and Likert scales, this instrument is based on a Q-sort method with cards that picture 18 life domains (see Figure 1). The card-sorting task helps patients with cognitive or verbal impairments to score their difficulties and needs on two 4-point Likert-type scales (level of difficulty and urgency of need for care). The ELADEB is widely used in French-speaking countries (Switzerland, France, and Canada) as “Echelles Lausannoises d’Auto-évaluation des Difficultés Et des Besoins” (21). A computerized version of the ELADEB has also been developed. This instrument can currently be used on smartphones and tablets with the benefit of instantly and automatically calculated scores.

Twelve items are identical to the CAN, four items are a combination of several items of the CAN (mental health, addiction, housework, and administrative tasks), and four items are new (work, public places, family, and friendship). The psychometric properties of this scale have been validated (21). The instrument is divided into two distinct subscales that are administered separately: (1) evaluation of difficulties and (2) evaluation of needs for additional intervention. The degree of urgency is coded into three levels: non-urgent needs (patients can wait for more than 3 months before an intervention), moderately urgent needs (the intervention is expected between 1 and 3 months), and urgent needs (the intervention is expected within 30 days). Patients are invited to sort out and rank the cards representing the domains, in which they perceive difficulties and needs. This method allows patients with pronounced cognitive or verbal impairments to make adequate subjective self-reports of their current problems and requests for additional care. Each of the 18 items score from 0 (no problem/need) to 3 (very important problem/urgent need). Clinical and rehabilitation needs can be assessed during the Q-sort even if they are not structured this way. For instance, rehabilitation needs could be expressed through the “treatment” item or by a more indirect, but nonetheless specific, manner (e.g., expressing needs for “mental health,” “work,” or “accommodation”). Quantitative scores are reported on an Excel^®^ file, which automatically creates an individual profile of the importance of difficulties and the urgency of needs illustrated with a bar chart. Therefore, 36 separate item scores and two global scores are derived from the Q-sorting task. For the purpose of this study, only the urgency of needs scores was used in the statistical analyses.

### Analysis

The two main factor analytic techniques used to assess the structure of psychometric scales are EFA and confirmatory factor analysis (CFA). EFA is used when the relationship between items and latent variables is uncertain or unknown, while CFA is used when the researcher has prior knowledge about the relationship between items and factors. Nevertheless, EFA is not totally exploratory because the number of factors to be retained must be determined and a method for factor rotation should be selected.

Many criteria have been proposed to determine the number of factors. Some of the better known methods are the Kaiser criterion (eigenvalue >1), the Cattell scree test, Horn’s parallel analysis, or Velicer’s minimum average partial method (MAP). Parallel analysis and MAP have proven to be the most accurate methods, and the Kaiser rule is considered as the most inaccurate (12). However, many of these criterions have been developed for continuous variables and are often inaccurate or not well suited for ordered categorical or binary measures. Since the items of ELADEB were categorical ordinal, we used a selection strategy based on both theoretical interpretability and goodness-of-fit indices. For that purpose, the comparative fit index (CFI) performs relatively better than the Tucker–Lewis index (TLI) and the root mean square error of approximation index (RMSEA) with categorical ordinal measures. A cutoff value close to 0.96 for CFI has acceptable rejection rates across models when the sample size is >250 (22).

Getting back to the question of the choice of the factor rotation, its type is orthogonal when researchers hypothesize that the factors are not correlated and oblique when it is assumed that factors are or could be correlated to each other (12).

Despite adequate model fit, EFA with conventional rotation methods (e.g., Promax and Varimax) did not give interpretable results. Other solutions with more usual rotation method corresponded to complex factorial structures, for which a meaningful interpretation of the factors was not possible.

This led us to reconsider our approach to assess the dimensionality of self-assessed needs. Let us remember that most rotation methods are designed to recover a simple and clean structure (e.g., each item loads on only one factor). However, more complex structures may also be hypothesized. When both unity and diversity are expected within the constructs measured by the items, bifactor models may be more adequate (23). The bifactor structure is characterized by one general factor and a number of group factors. The general factor represents the commonality between the items, and the specific factors account for the unique influence of specific domains over the general factor. In this case, items can reflect both general and more specific constructs. We chose the oblique bi-geomin method, which is an EFA rotation criterion that allows all items to load directly onto a general factor and also onto one specific factor (14). The general factor is modeled orthogonally to other factors and a perfect cluster structure for the loadings on the remaining factors is sought. The specific factors could be correlated among themselves (23). It allowed us to explore whether needs would show both unity and diversity and if derived factors would be more interpretable.

All models were estimated using a robust weighted least squares estimator with adjustments for the mean and variance (WLSMV). All statistical analyses were performed with the Mplus 7.0 software (24).

## Result

Table 2 shows the distribution of need ratings on the four levels (from 0 = no need to 4 = urgent need). The three domains, where the most additional help was needed, were “mental health” (45.9%), “finances” (37.6%), and “work” (37.6%). In other domains, 70–85% of patients reported no need.

**Table 2:** **Univariate proportions and frequencies for need for care (*N* = 471)**.

Rating								
Items	No need for care		Non-urgent need (more than 3 months)		Moderately urgent need (between 1 and 3 months)		Urgent need (within 30 days)	
	%	Count	%	Count	%	Count	%	Count
Accommodation	76.01	358	5.10	24	8.07	38	10.83	51
Finances	62.42	294	8.49	40	9.98	47	19.11	90
Work	62.42	294	10.19	48	14.01	66	13.38	63
Free time	83.44	393	4.67	22	5.10	24	6.79	32
Administrative tasks	75.16	354	6.79	32	8.70	41	9.34	44
Housework	85.35	402	2.55	12	6.58	31	5.52	26
Transports	84.93	400	4.67	22	6.16	29	4.25	20
Public places	84.08	396	3.61	17	7.86	37	4.46	21
Friendship	78.98	372	5.52	26	7.01	33	8.49	40
Family	80.25	378	5.52	26	8.70	41	5.52	26
Children	85.77	404	2.97	14	4.88	23	6.37	30
Intimate relationships	77.49	365	8.70	41	6.58	31	7.22	34
Food	77.92	367	5.31	25	5.10	24	11.68	55
Self-care	94.27	444	1.70	8	1.70	8	2.34	11
Physical health	71.76	338	5.73	27	9.77	46	12.74	60
Mental health	54.14	255	7.86	37	12.10	57	25.90	122
Addiction	84.50	398	5.52	26	4.67	22	5.31	25
Treatment	75.16	354	4.03	19	5.73	27	15.07	71

The other columns indicate the urgency of needs for each dimension. When needs were expressed, patients tended to indicate moderate or urgent needs more frequently than non-urgent needs. More specifically, the frequency rating for non-urgent needs was generally equal or inferior to the frequency rating for moderately urgent needs except for “intimate relationships” (8.7% non-urgent needs, 6.6% moderately urgent needs, and 7.2% urgent needs).

Non-urgent need (possibility to wait for more than 3 months before an intervention) was chosen by more than 10% of the patients only in the “work” domain. “Mental health” and “finances” were the two most often reported items regarding urgent need (25.9 and 19.1%, respectively).

Urgent needs were expressed by more than 10% for the “treatment” (15.1%), “work” (13.8%), “physical health” (12.7%), “food” (11.6%), and “accommodation” (10.8%) domains. Most of the expressed needs were considered urgent by patients except for the “work” domain. This shows a contrast between short- and long-term concerns. Patients reported short-term concerns for health and finances, but long-term concerns regarding work.

Solutions including one to four factors were compared. As shown in Table 3, the criteria of adequate fit were first met with the three-factor model (CFI = 0.970). Comparison between the three- and four-factor solutions revealed that the general and the first two specific factors were similar. Nevertheless, the two specific factors were much more clearly defined in the four-factor solution. As the interpretation of the extra specific factors was meaningful and straightforward, we chose to report the four-factor solution (Table 4). Because the goodness-of-fit for the four-factor model was excellent, no further factors were extracted.

**Table 3:** **Comparisons of model fit for need for care**.

Model	**χ**^2^	df	RMSEA	Probability RMSEA **≤**0.05	SRMR	TLI	CFI
Single-factor model	279.294	135	0.048	0.679	0.094	0.896	0.908
Two-factor model	212.069	118	0.041	0.951	0.078	0.923	0.940
Three-factor model	149.878	102	0.032	0.999	0.062	0.954	0.970
Four-factor model	111.081	87	0.024	1.000	0.052	0.973	0.985

*df, degree of freedom; RMSEA, root mean square error of approximation; SRMR, standardized root mean square residual; TLI, Tucker–Lewis index; CFI, comparative fit index*.

**Table 4:** **Factor loadings for the exploratory four-factor model of needs for care (oblique bi-geomin rotation)**.

Items	Factor loadings			
	General	F1 – management	F2 – functional disabilities	F3 – familial and interpersonal relationship
Accommodation	**0.449**	0.205	**−**0.135	**−**0.047
Finances	0.290	**0.981**	0.029	0.028
Work	**0.459**	0.055	**−**0.323	**−**0.045
Free time	**0.682**	**−**0.126	0.232	0.070
Administrative tasks	**0.470**	**0.311**	**−**0.098	**−**0.144
Housework	**0.494**	0.088	**0.346**	**−**0.015
Transports	0.277	0.178	**0.497**	**−**0.032
Public places	**0.579**	**−**0.085	**0.453**	0.031
Friendship	**0.549**	**−**0.063	0.182	**0.363**
Family	**0.655**	0.083	**−**0.024	**0.443**
Children	**0.543**	0.188	**−**0.086	**0.434**
Intimate relationships	**0.609**	**−**0.095	0.037	**0.395**
Food	**0.578**	**−**0.010	**0.293**	**−**0.260
Self-care	0.352	0.031	**0.370**	**−**0.146
Physical health	**0.609**	**−**0.004	**−**0.009	**−**0.263
Mental health	**0.792**	**−**0.039	**−**0.130	0.018
Addiction	0.363	**−**0.065	**−**0.154	**−**0.155
Treatment	**0.634**	**−**0.102	**−**0.003	0.054

	Factor correlations			
	General	F1 – management	F2 – functional disabilities	F3 – familial and interpersonal relationship

General	1.000			
F1	0.000	1.000		
F2	0.000	**−**0.089	1.000	
F3	0.000	**−**0.020	**−**0.059	1.000

*Loadings in bold are <0.30*.

Results showed a general factor with loadings >0.40 on every item except “finances,” “transports,” “self-care,” and “addiction.” The “finances” item showed specificity by loading uniquely on the first specific factor (0.98). “Transports” and “self-care” loaded on the second specific factor (0.50 and 0.37, respectively). The “addiction” item did not load on any specific factor and only moderately on the general one (0.36). So each item was clearly represented in the structure except one (“addiction”).

The first specific factor was essentially composed by the financial needs. “Administrative tasks” also loaded moderately on the first specific factor (0.31). The second specific factor was mainly defined by “transports” and “public places,” with moderate loadings for “self-care” (0.37), “housework” (0.35), and “food” (0.29). Finally, the “family,” “children,” and “intimate relationships” items defined the last and third specific factor. “Friendship” also loaded moderately on this third specific factor (0.36). Familial and relational needs tended to be expressed altogether by the patients.

The correlations between the general and specific factors are also shown in Table 4. Generally, the four factors were weakly correlated to each other. The specific factors can be interpreted as the unique influence of specific domains over the general factor. They were not related to each other. This means that patients requesting specific help in one domain did not report needs in other specific domains.

## Discussion

This study is the first to investigate the factorial structure of patients’ needs for intervention with the bifactorial approach. This method led us to identify a structure of patients’ needs with four factors: one general and three specific factors. This four-factorial structure offers a well-defined place for every item, except “addiction.”

A general factor can be interpreted as the domain-general component of every item (with significant loadings). It represents unidimensionality in needs for care. The general factor shows patients’ tendency to have either urgent or non-urgent needs in every life domain. Therefore, the general factor tends to distinguish patients’ needs more in terms of urgency than in specific life domains. Patients reporting urgent needs in one specific domain tend to report the same level of urgency in other domains. Therefore, the level of urgency is not specific to one particular domain. Similarly, patients reporting non-urgent needs tend also to report non-urgent needs in other domains. Thus, the general factor can be considered as an indicator of crisis for the patient more than an indicator of one specific need domain.

By contrast, the three specific factors indicate the existence of independent dimensions beyond the global urgency of need. They may be viewed as additional components of needs and are used to explain departures from unidimensionality. They cover patients’ needs in three domains: (1) management, (2) functional disabilities, and (3) familial and interpersonal relationships. The first factor shows essentially the presence of financial but also administrative needs (patients struggling with finances also tend to seek help regarding administrative tasks but not systematically). “Transports” has the highest loading on the second specific factor, which also comprises “public places.” We could have first interpreted this factor as an “agoraphobia” factor because these types of needs are related to difficulties in going out to public places, as well as using public transportation. The positive saturation of the “self-care” item found on this factor is also compatible with such an interpretation. Indeed, patients who were conscious of their personal hygiene needs may be more reluctant to go outside and interact with others. However, as “housework” and “food” also positively load on this factor, even if less strongly, and because agoraphobia implies a very specific meaning, we preferred using a more global name, such as “functional disability.” The third factor, which is labeled “interpersonal relationships,” comprises “family,” “children,” “intimate relationships,” and “friendship” (with a moderate loading on it).

As mentioned in Section “Introduction,” the number and types of factors vary between the studies dedicated to the measurement of patients’ needs. Korkeila et al. (9) used the CAN and obtained four factors, whereas Salvi et al. (10) found seven factors with the CANSAS. When needs are self-evaluated by patients, the factorial structures are, again, divergent: Korkeila et al. (9) obtained five factors, whereas Ritsner et al. (11) used the CANSAS-P and obtained four factors. However, all of these studies tend to show specific types of needs for intervention expressed by patients. In some aspects, our results are similar, and in others, different from those found in the literature. The major novelty obtained within this current study lies in the identification of a general factor. This general factor has never been reported in previous studies. Indeed, the factorial structures found in the literature are all organized around specific types of needs for intervention. This general factor can be considered as an indicator of crisis for the patient indicating the current level of urgency of needs.

Regarding the specific factors we identified, one study obtained a similar structure to our results (8). These researchers used the CAN rated by key workers with patients suffering from severe mental illness. They obtained a fairly simple structure that separates (1) functional disability, (2) social loneliness, and (3) emotional loneliness. Our results split the functional disability factor proposed by Wennström et al. (8) in two dimensions. Indeed, our findings distinguish “management needs” (comprising “finances” and “administrative tasks”) from “functional disabilities” (comprising “transports,” “public places,” “self-care,” “housework,” and “food”). On the contrary, their social and emotional loneliness factors are combined into one “interpersonal relationships needs” factor in our results. Such differences could be explained by differences in the content of the instruments. However, because general factors are typically not represented with more classical EFA, the unique influence of specific domains over the general factor is in fact still largely unknown in other studies. Previously identified specific factors could also differ from the present study because variance from the general factor was not partialed out (23). Based on our interpretation of the general factor as a possible crisis indicator, we emphasize the usefulness of disentangling the influence of specific domains over the general factor. We hypothesize that with other instruments the structure of needs could also show both unity and diversity (i.e., bifactorial structure).

### Limitations

Several methodological limitations can be identified. This study reported the results of patients’ self-evaluations of needs. If the self-reported needs of patients seem essential in planning treatment strategies adapted to patients’ needs, it would be interesting to compare and contrast these perceptions with staff ratings. This should ideally be performed with two different and independent investigators in order to avoid patients’ perspectives being filtered by staff or vice-versa (18). As the ELADEB is different from the CANSAS, it would be interesting to use both measures with the same sample of patients. Such a protocol could provide validation data for both instruments and permit a real direct comparison between them.

The second major limitation of our study is that data on patients’ social functioning, chronicity, level of depression, or anxiety, etc., are missing. Adding these data would allow for the assessment of more specific questions, such as the role of chronicity in the profile of patients’ needs.

One should also note that model fit in EFA is not affected by the rotation method. It was therefore not possible to compare different solutions based on the same number of factor but on different rotation methods on statistical grounds. This means that our work was not purely empirical but also highly theory-driven. Indeed when evaluating the quality of a model one cannot simply rely on statistical results and must also evaluate how interpretable and useful a given model is in light of the substantive phenomenon under investigation.

### Clinical Implications

This study has two levels of implications: an individual and a public mental health level. On an individual level, the specific factors obtained showed the specific domains, in which patients experience needs for care. For example, if a patient tends to have needs regarding his family life, he probably will also experience needs in terms of other interpersonal relationships, such as friendship. The specific factors we found relate mainly to social interventions (finances, functional disability, and interpersonal relationships), which are generally less covered by classical psychiatric or psychotherapeutic interventions. These domains seem to be more covered by rehabilitative or community interventions, such as competences training, financial help, or help for social integration. Interestingly, the three main areas of specific needs are similar to the four types of social support classically reported in the literature: financial, emotional, self-esteem, and instrumental support (25–27). It is interesting to think about a relationship between the types of specific needs expressed by patients and the kinds of social support found and described in the literature. Therefore, our results and model is coherent when related to this social support model.

Moreover, the concept of need, defined as the degree of urgency for additional intervention, facilitates an immediate health care response that is adapted to the patients’ perception of their needs. As a function of the patients’ perceptions, the clinical care team can immediately orient and develop further interventions responding and corresponding to the degree of urgency.

Another important clinical implication related to this individual level relies on the fact that the ELADEB is entirely based on patients’ perspectives. There is now considerable evidence that clinicians tend to consistently misread their patients’ needs and wants, while confidently considering that they rate them correctly (28, 29). A patient-centered psychiatric practice facilitates empowerment, as it is based on the patients’ personal motivations, needs, and wants (20, 30, 31). Furthermore, including the patient’s point of view enhances the clinician–patient relationship (32). The use of a self-assessment scale represents a unique way of obtaining patients’ perceptions. Therefore, these results have clinical implications in terms of better understanding patients’ functioning according to the different domains of their needs for care. These results may be beneficial in order to use adapted treatment strategies according to the patients’ specific and personal needs for care. Furthermore, self-evaluations should be done with tools that are easily understood by patients with chronic mental illnesses, they should also be “pure” in terms of avoiding interferences from the staff’s perspective of the patients’ needs. Therefore, the ELADEB is based on a Q-sort method using cards with pictures that facilitate self-evaluation. Consequently, this instrument can easily be adapted and translated into different languages. The computerized version for smartphones or tablets is as easy and intuitive to use as the printed version and shows immediately the results in a table and a graph. Furthermore, this tool is adapted to the clinical context and to patients with chronic cognitive or verbal deficits. At the same time, it is based on scientific standards with validated psychometric properties.

On a public mental health level, our findings indicate that some patients demonstrate an urgent need for intervention in almost every domain. Other patients may report a similar level of symptoms but do not seek intervention services as often. These results address, therefore, the question of high consumers and users of mental healthcare versus low users. High consumers may mobilize most of the resources in mental healthcare services. From a public mental health standpoint, it is therefore beneficial to be attentive to and aware of this general factor showing that patients tend to have either urgent needs in different areas or non-urgent needs in several domains. This phenomenon of unity versus diversity of needs for care is therefore relevant at both the individual and the public mental health level. Still from a public health perspective, ELADEB may be proved worthwhile to characterize case-mix. Identifying self-reported needs at both the individual and institutional level may guide decision-making in regard to the allocation and resource use. While needs assessment is recognized to be a central element of mental health care, staff assessment, and self-assessment of patients’ needs diverge. Thus, identifying important differences between admission and discharge could complement traditional routine clinical outcome measures.

## Conclusion

This study provides a synthesis and structure of patients’ needs for care using a recent self-assessment scale as well as a recently published statistical method. It offers a new perspective in suggesting a four-factor model including one general and three specific dimensions of needs that represent management, functional disabilities, and familial and interpersonal relationships.

## Author Contributions

TB-T: database creation; redaction of introduction, method, results, and discussion; and revision and final modifications. PG: statistical analyses; redaction of method: statistical analyses; and revision and final modifications. CB: revision and final modifications. VP: revision and final modifications.

## Conflict of Interest Statement

The authors report no conflict of interest. The authors alone are responsible for the content and the writing of this article.
